# Influence of high altitude on the burning behaviour of typical combustibles and the related responses of smoke detectors in compartments

**DOI:** 10.1098/rsos.180188

**Published:** 2018-04-04

**Authors:** Ran Tu, Yi Zeng, Jun Fang, Yong-Ming Zhang

**Affiliations:** 1 College of Mechanical Engineering and Automation, Huaqiao University, Xiamen, Fujian 361021, China; 2 Key Laboratory of Process Monitoring and System Optimization for Mechanical and Electrical Equipment, Fujian 361021, China; 3 College of Tourism, Huaqiao University, Quanzhou, Fujian 362021, China; 4 State Key Laboratory of Fire Science, University of Science and Technology of China, Hefei, Anhui 230026, China

**Keywords:** high altitude, EN54 test fires, burning rate, smoke detector, response signal

## Abstract

The effect of altitude on typical combustible burning and related smoke detector response signals was investigated by comparison experiments at altitudes of 40 m and 3650 m based on EN54 standard tests. Point-type light scattering photoelectric smoke detectors and ionization smoke detectors were used for four kinds of EN54 fire tests, including two kinds of smouldering fires with wood (test fire no. 2 in EN54 standard or TF2) and cotton (TF3), and two kinds of flaming fires with polyurethane (TF4) and *n*-heptane (TF5). First, the influence of altitude or ambient pressure on mass loss for smouldering combustion (TF2 or TF3) was insignificant, while a significant decrease in the mass burning rate was found for flaming tests (TF4 and TF5) as reported in our previous studies. Second, for photoelectric smoke detectors in flaming fire tests, the effect of altitude was similar to that of the burning rate, whereas for the ionization smoke detectors, the response signal at high altitudes was shown to be ‘enhanced’ by the detection principle of the ionization chamber, leading to an even larger value than at normal altitude for smouldering conditions. Third, to provide a reference for smoke detector design in high-altitude areas, the differences between signal speed in rising and peak values at two locations are discussed. Also, relationship between ion chamber signals and smoke optical densities are presented by utilization of an ionization smoke detector and smoke concentration meter. Moreover, a hierarchical diagram is illustrated to provide a better understanding of the effects of altitude on combustible burning behaviour and the mechanisms of detector response.

## 1. Introduction

Interest in fire detection engineering in high-altitude areas stems from the need to protect both the local populace and historic buildings. One such area is the Qinghai-Tibet Plateau in China, with an average altitude of 4 km. It is called the roof and the third pole of the world and contains thousands of historic buildings, including the Potala Palace. Environment parameters such as humidity, air pressure and temperature can significantly influence fire dynamics and burning behaviour [1–13]. For example, with increasing altitude, the decreasing ambient pressure and the absolute concentration of oxygen (e.g. in Lhasa, Tibet, the pressure is approximately 2/3 atm) have been shown to slow down the burning rate of liquids and solid fuels (e.g. [5,8,12,13]). Also, flame or plume physical characteristics and temperature profile have been proved to be affected by pressure obviously [7,9–11]. Consequently, smoke formation, concentration and movement would also change, which introduces new challenges for smoke detectors used in these areas.

Burning rate is one of the key parameters in the fire combustion process and also an important variable used to determine smoke behaviour. The previous experimental studies about pressure effects on the burning rate of wood [5,8], flexible polyurethane [12], *n*-heptane [6,7,9] etc. showed a complex tendency. In our recent study [13], an overall correlation between altitude or pressure effects and burning rate was theoretically derived based on pressure modelling [14] and radiation fire modelling [15]. This correlation could provide an effective prediction of the burning rate variation for liquid fuels at different altitudes.

Smoke detectors, mainly including the line and point types, are widely used for fire detection. Early investigations of altitude effects on the response of smoke detectors were reported by Wieser [2], who conducted four kinds of fire tests at four different altitudes with pressures ranging from 715 to 976 mbar. They used a scaled EN54 test [16] chamber with dimensions of 6 m × 2.8 m × 2.1 m instead of the standard test room, accounting for the necessary transportation between altitudes. The optical extinction value *m* and the ionization chamber value *y* were compared, and the results showed that the smoke concentration under the ceiling decreased significantly with increasing altitude. This indicates that a smoke detector may trigger an alarm in a timely manner at normal altitudes but may not do so at high altitudes, even for the same fire source scenario.

**Figure 1.**
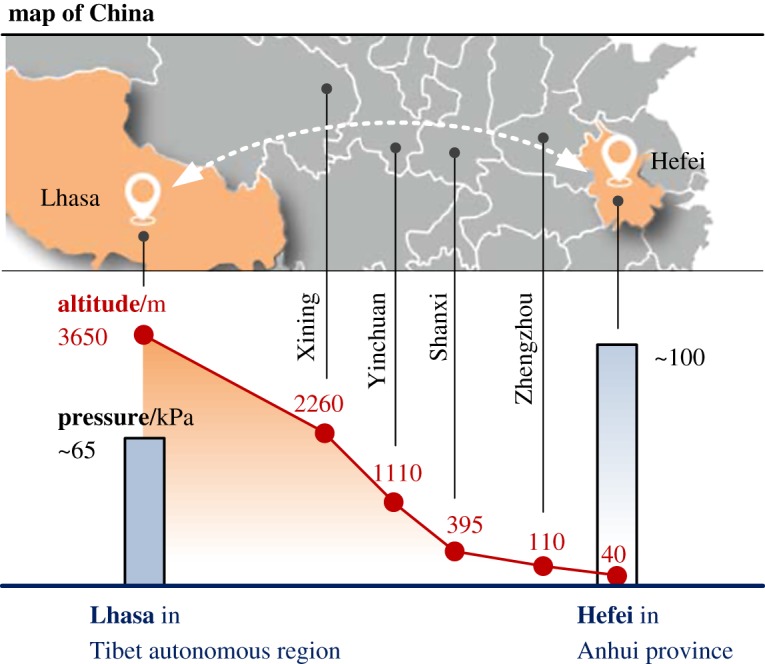
Comparison of altitudes and air pressures between Lhasa and Hefei (including another four altitudes of provincial capitals).

In this study, comparative fire tests based on the EN54 test standard [16] were performed in Lhasa (air pressure 65.0 ± 1.5 kPa with an altitude of 3650 m) and Hefei (air pressure 100.0 ± 1.0 kPa with an altitude of 40 m), two representative locations in China shown in figure 1. Two ordinary kinds of point-type smoke detectors, photoelectric smoke detectors (Pho smoke detectors) based on light scattering and ionization smoke detectors (Ion smoke detectors) based on measuring the ionization chamber, were employed to study the influence of altitude on the variation of fire smoke characteristics and detector response rules. The aim of this study is to provide a useful reference for smoke detector design and fire protection engineering at high-altitude conditions.

## 2. Methods

### 2.1. Facility and instruments

Comparison experiments were conducted inside the same two EN54 standard test rooms with dimensions of 10 m long, 7 m wide and 4 m high, built in Lhasa and Hefei. The air temperature and humidity inside the test rooms (2 ± 2.0°C, 50 ± 5%) were controlled by air conditioning to eliminate added interference.

**Figure 2.**
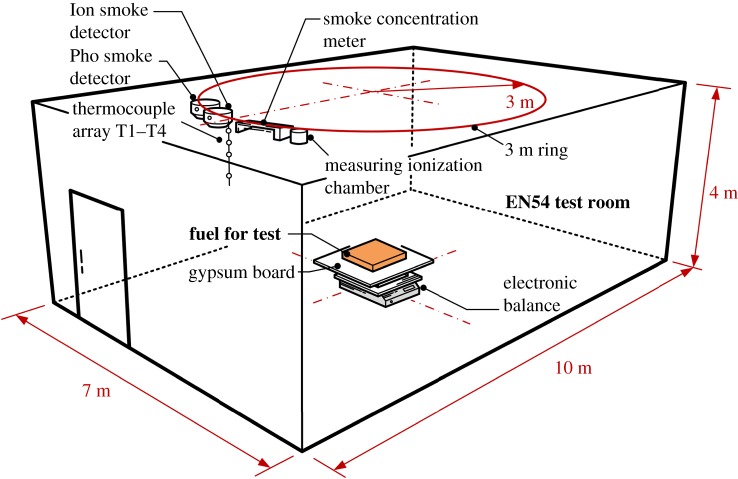
Sketch of the experimental facility with a perspective view.

As shown in figure 2, fire sources with different fuels were set in the centre of the floor in each EN54 test room. Thick gypsum boards were used to protect the electronic balance beneath the boards (Excellence-Plus XP with precision of 0.01 g by Mettler Toledo Co. Ltd, Switzerland), which recorded the fuel mass variation online. Two point-type smoke detectors, including one Pho smoke detector (GD 2000 by EI Fire Protection Co. Ltd, China) and one Ion smoke detector (M 2000 by EI Fire Protection Co. Ltd, China), were mounted at the 3 m ring [16] under the ceiling. The response signals of the two detectors were recorded using an acquisition module, which indicated the strength of the smoke concentration with a range from 0 to 255 (with unit: 1). Smoke concentration meters (AML by Lorenz Ltd, Germany) and ionization chamber meters (EC-912 by DELTA Ltd, Denmark) were also fixed at the 3 m ring near the smoke detectors. Smoke layer temperatures were measured using Type K armoured thermocouple arrays with a diameter of 0.5 mm and an uncertainty level of 0.75% and thermocouples T1–T4 in positions located 4 cm, 20 cm, 40 cm and 70 cm under the ceiling, respectively. An HD digital camera (with frequency of 30 fps by Sony Co. Ltd, Japan) was used to monitor the fuel burning behaviour and smoke movement. Any trade name is used for descriptive purposes only.

### 2.2. Experimental procedure

**Figure 3.**
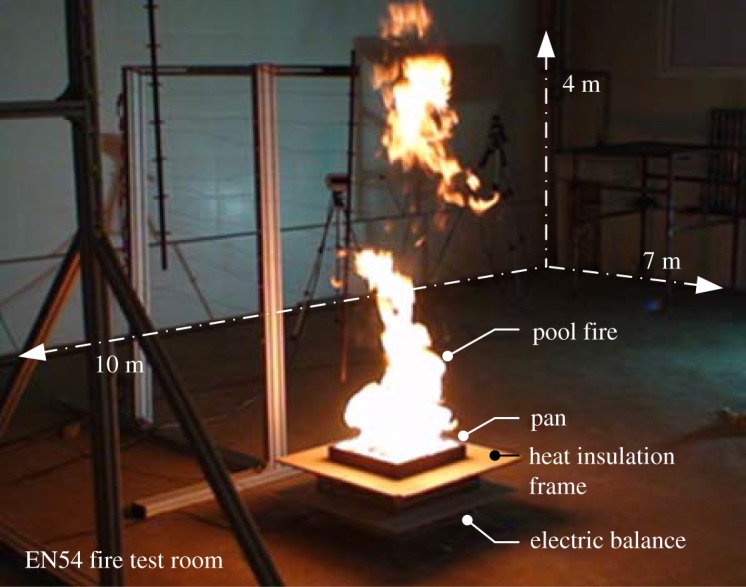
Interior view of the TF5 test in the EN54 fire test room.

Four EN54 test fire sources TF2–TF5 [16] (two kinds of smouldering fires and two kinds of flaming fires) were selected as illustrated in table 1 for comparison experiments. The detailed test procedures were based on the arrangement described in the EN54 test method, which is only introduced briefly here. The temperature and humidity inside the room were adjusted before the start of the test. The response signal of the smoke detectors, the smoke concentration and the temperature of each measuring sensor were recorded throughout the entire test online, which could also be monitored from a display terminal in the control room. Tests were repeated to ensure results with high accuracy. A typical experimental scenario is shown in figure 3.

**Table 1.** Test fires used for comparison experiments.

test fire	explanation	mass	fuel explanation
TF2	smouldering wood fire	150 g	beechwood sticks, heated by electrical hotplate with 2 kW
TF3	smouldering cotton fire	270 g	cotton wicks, ignited from the bottom
TF4	polyurethane flaming fire	620 g	50 cm long, 50 cm wide and 6 cm thick, ignited from one corner
TF5	*n*-heptane flaming fire	650 g	burnt in square steel pool 33 cm long, 33 cm wide and 5 cm deep

## 3. Results and discussion

### 3.1. Mass variation and the response of detectors in smouldering conditions

**Figure 4.**
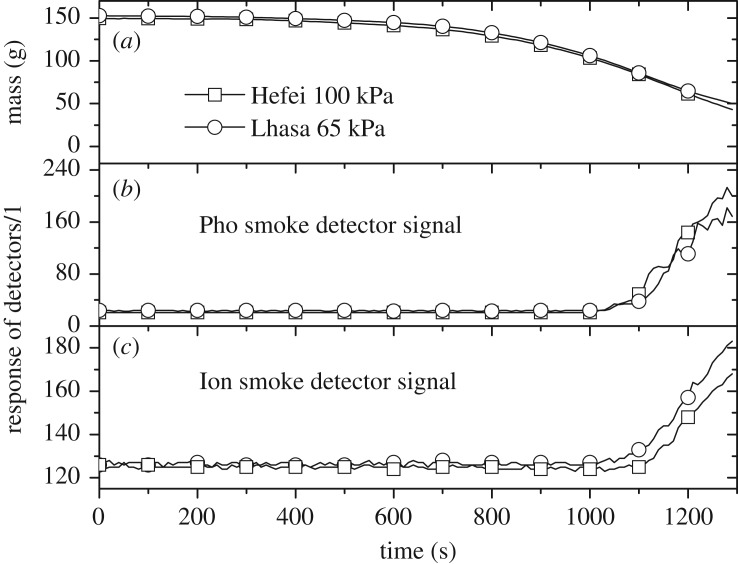
Comparison of TF2 mass variation (*a*), and response signals of Pho smoke detectors (*b*) and Ion smoke detectors (*c*) between the two locations.

The comparison of the burning rate and smoke detector signal for TF2 (wood) is shown in figure 4. The background values (initial constant value without smoke) for the two kinds of detectors are different due to the different signal processing mechanisms. It can be noted that the influence of altitude on the burning mass loss of smouldering wood is negligible, as indicated by the limited oxygen consumption rate in the smouldering condition, which is much less than that in the flaming combustion condition. In addition, the actuation times (when the signal started rising) of both Pho and Ion detectors, which started around 1000 s, are shown to be seriously delayed compared to the mass loss shown in figure 4*a*. This was caused by the slow smoke movement driven by low temperature buoyancy, which made it even more difficult for the smoke to reach the ceiling due to the cooling effect of the ambient cold air. The thermocouple array showed that, in the later stages of smouldering, the temperature of the smoke layer is only approximately 2°C higher than initial ambient temperature. The response of the Pho smoke detector in Hefei shows it to be slightly higher than that in Lhasa, whereas the Ion smoke detector shows an opposite tendency, which will be discussed later.

**Figure 5.**
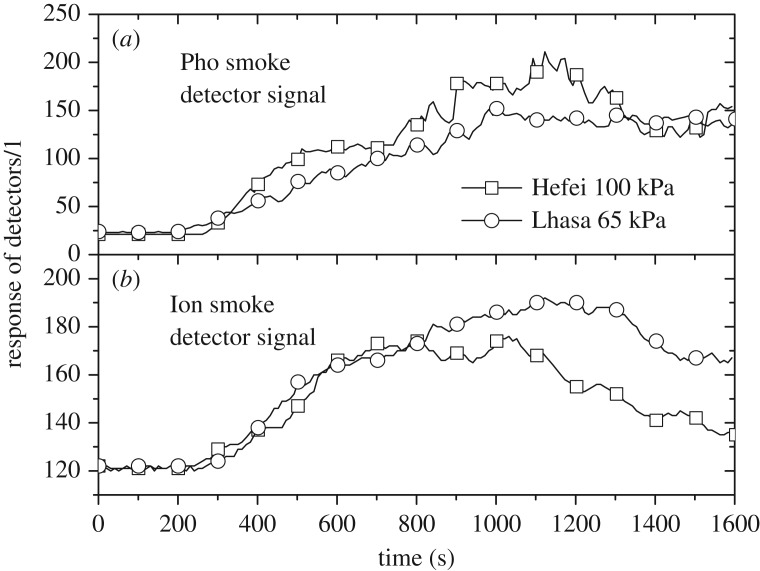
Comparison of TF3 response signals of Pho smoke detectors (*a*) and Ion smoke detectors (*b*) between the two locations.

TF3 (cotton) test results were similar to those of TF2. The smouldering burning rates between the two altitudes were about the same, and figure 5 shows the comparison of smoke detector signals. Because of the larger smouldering burning area in the early stage for TF3 than TF2, the smoke movement was faster, driven by the larger heat release rate from TF3, resulting in a shorter actuation time of approximately 200 s. For the smoke detector responses, similar to the results for TF2, Pho smoke detectors in Hefei show higher levels than those in Lhasa, while the opposite is true for Ion smoke detectors.

### 3.2. Mass burning and the response of detectors in flaming conditions

**Figure 6.**
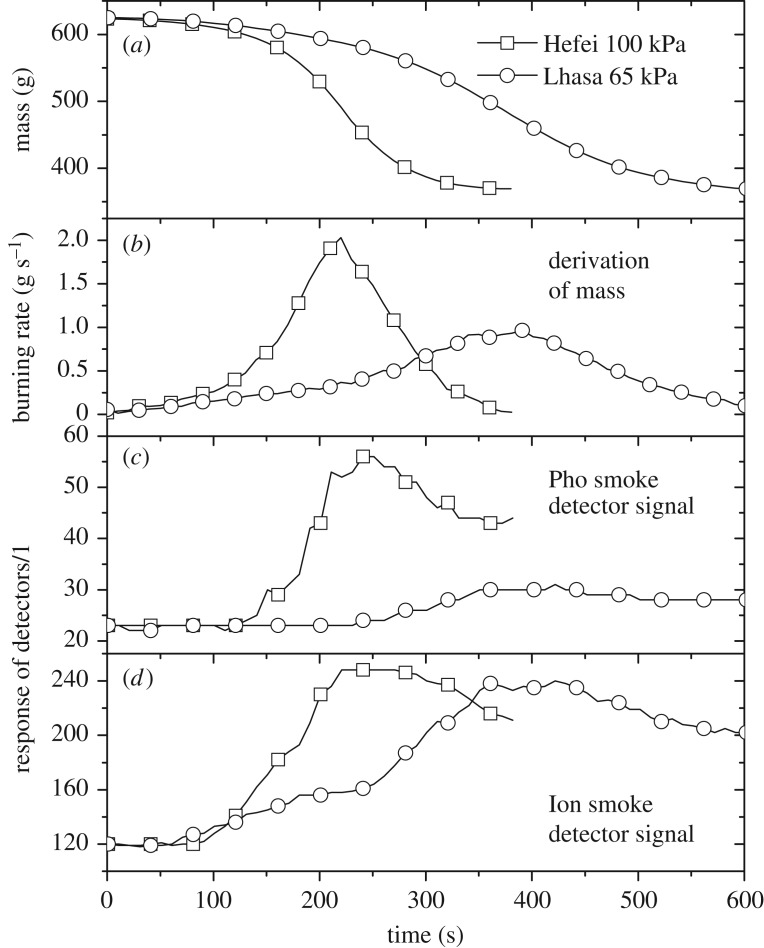
Comparison of the TF4 mass variation (*a*), burning rate (*b*), and response signals of Pho smoke detectors (*c*) and Ion smoke detectors (*d*) between the two locations.

The results of flaming fires were completely different from the results of the smouldering conditions. Test data of TF4 (polyurethane) are plotted in figure 6. As reported in our previous studies [9,12,13], the influence of altitude on the flaming burning rate $\dot{m}$ is complicated, as shown in figure 6*a*,*b*, due to the pressure effects on the flame heat feedback mechanism ${\dot{m}}^{\prime \prime }\propto p^{\alpha }$ (*α* varies from about 0 to 2 depending on the burning scale). One of the main effects is that the soot formation rate per volume $\bar{{m^{\prime \prime \prime }}_{s}}$ under low-pressure conditions becomes much slower in high altitudes as $\bar{{m^{\prime \prime \prime }}_{s}}∼p^{2}$ [12,13,15], causing fewer soot particles and less radiation heat feedback.

Figure 6*c* shows that the actuation of detectors was much faster than that for smouldering tests, which is attributed to the strong turbulent buoyancy driven by the relatively large heat release in flaming burning. From the video monitor, it could be seen that the smoke could get to the ceiling a few seconds after burning started. Considering that the light scattering strength is proportional to particle number density $\mathrm{LS}∼\sum n_{i}d_{i}^{2}$ (where *n* is the number of particles and *d* is the particle diameter; subscript *i* indicates the particle size distribution) [17,18], this explains why the signals of Pho smoke detectors showed the same trend with burning rates, as shown in figure 6*b*,*c*.

It was interesting to note that although the signal strength of the ionization chamber should also be proportional to soot particle number density, the Ion smoke detector signal in Lhasa, shown in figure 6*d*, showed a significant increase compared to the curve of the Pho smoke detector signal in Lhasa, shown in figure 6*c*. Now we return to the question left in the TF2 test regarding the different behaviours of Pho and Ion smoke detectors for both smouldering and flaming burning. The main reason for this difference stems from the detection principle of the ionization chamber in smoke detectors, which is based on the fact that radioactive sources will increase the ability of the air to conduct electricity, which is also affected by altitude and air pressure. The initial number of ionized air particles, or initial current *I* of the ionized air inside the Ion smoke detector chambers is hence smaller at high altitudes. Because the response signal of Ion smoke detectors is proportional to Δ*I*/*I* (where Δ*I* is the current variation caused by soot particles entering the chamber and colliding with ionized air particles), the signal value is determined by the competing effects of decreasing Δ*I* and decreasing *I* at high altitudes. For the smouldering conditions in TF2 and TF3, the decrease in *I* is faster than the decrease in Δ*I* due to the limited influence of pressure on smoke soot production. The response at high altitudes would be even stronger, as shown in figures 4*c* and 5*b*.

**Figure 7.**
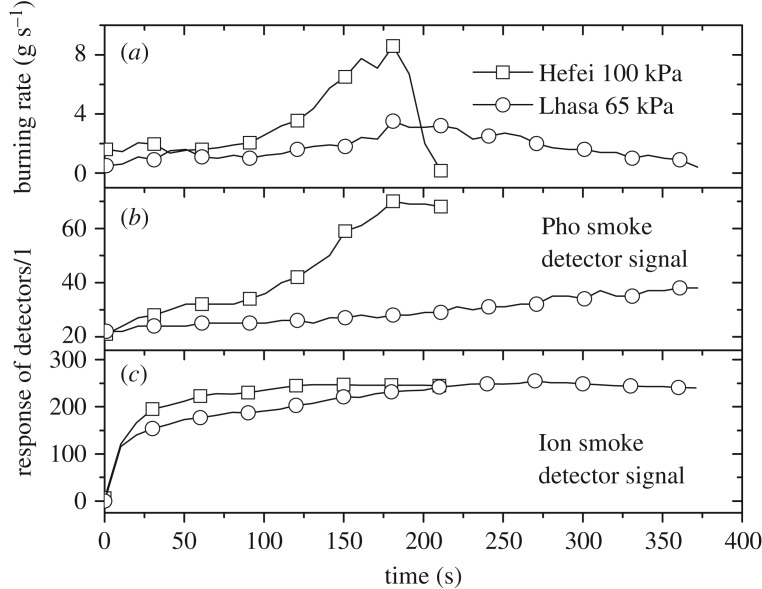
Comparison of the TF5 burning rate (*a*), and response signals of Pho smoke detectors (*b*) and Ion smoke detectors (*c*) between the two locations.

Figure 7 shows the test results of TF5 (*n*-heptane), which is a liquid pool fire with a larger heat release rate than TF4. Similar to TF4, the influence of altitude on Pho and Ion smoke detectors was also different. It can be noted that the burning rate in Lhasa decreased gradually at the later stages and that both detectors' signals persisted due to the maintenance of the hot smoke layer.

### 3.3. Comparisons of soot particle size distributions

**Figure 8.**
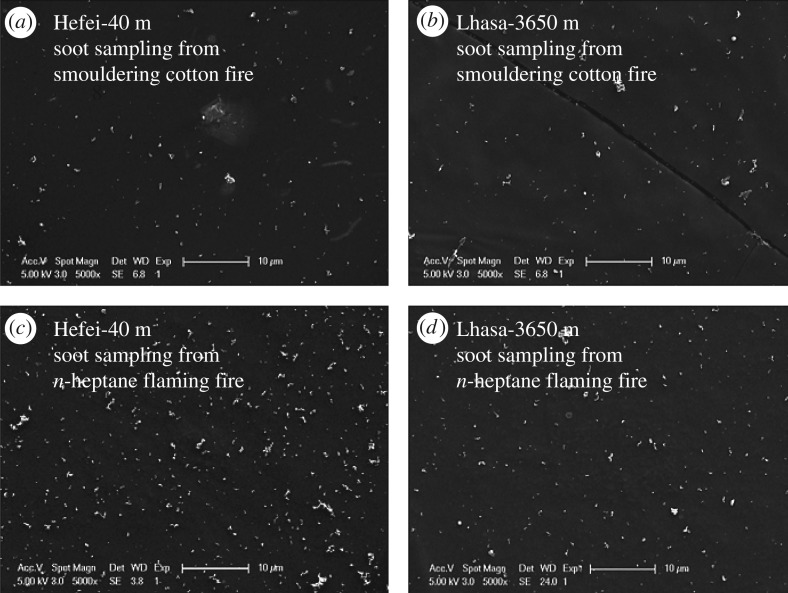
Comparison of SEM images of soot particles from smouldering cotton under normal pressure (*a*) and low pressure (*b*), and *n*-heptane flaming fire under normal pressure (*c*) and low pressure (*d*).

Considering the strong relationship of smoke detector sensitivity versus soot size [19] and the possible influence of altitude on soot size, cupreous web sampling of smoke soot and scanning electron microscopy (SEM) technique were employed to study the soot particle size distribution by a digital image process. Figure 8 shows the SEM images of soot particles from smouldering cotton (TF3) and an *n*-heptane flaming fire (TF5) under two altitude conditions with magnification 5000.

**Figure 9.**
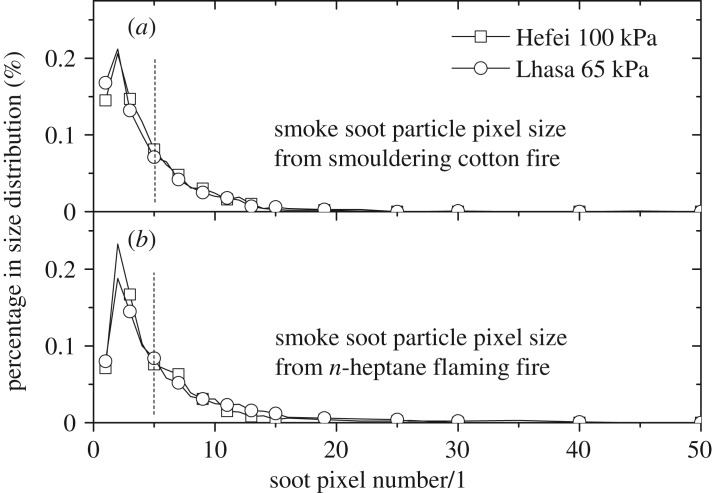
Comparison of soot particle size distribution of smouldering cotton (*a*) and *n*-heptane flaming fire (*b*) at both altitudes.

Soot pixel size distributions using the digital image process are plotted in figure 9 for a better comparison. It could be noticed that the pressure effect on soot size distribution is very limited. On the other hand, soot particles from smouldering cotton seem smaller than those from the *n*-heptane flaming fire as the soot pixel distribution centre is closer to the left-hand side. The reason for this phenomenon was that the burning scale rate and heat release rate of flaming fires were much larger than those of smouldering fires in EN54 tests, resulting in more primary soot particles and larger soot concentration. During smoke movement, the coagulation of soot from a flaming fire should be more significant due to faster colliding velocity and frequency, leading to relatively larger soot size.

### 3.4. Comparisons of detector signal characteristics

To obtain the response rules of smoke detectors at different altitudes, we compared the signal characteristics, e.g. averaged speed in rising and peak values, for each test fire in both locations. The upward phase of the response signal data was used to calculate the average rise in speed for Pho and Ion smoke detectors as given in table 2, with the speed rise defined as

3.1 $$\text{speed rise}=\frac{0.8\Delta S}{t_{0.9\Delta S}-t_{0.1\Delta S}},$$

where Δ*S* is the increment of maximum value of detector signal minus the initial value, and *t*_0.9Δ*S*_ and *t*_0.1Δ*S*_ are the time reaching 0.9Δ*S* and 0.1Δ*S*, respectively. Table 3 presents the comparison of peak values for different detectors.

**Table 2.** Comparison of averaged speed rise of detector signals.

	Pho speed rise (s^−1^)			Ion speed rise (s^−1^)		
test	Hefei	Lhasa	ratio	Hefei	Lhasa	ratio
TF2 (wood)	0.74	0.69	1 : 0.93	0.23	0.26	1 : 1.13
TF3 (cotton)	0.20	0.18	1 : 0.90	0.08	0.08	1 : 1.00
TF4 (polyurethane)	0.23	0.05	1 : 0.22	0.94	0.40	1 : 0.42
TF5 (*n*-heptane)	0.26	0.04	1 : 0.15	1.07	0.60	1 : 0.56

**Table 3.** Comparison of peak values of detector signals.

	Pho peak value/1			Ion peak value/1		
test	Hefei	Lhasa	ratio	Hefei	Lhasa	ratio
TF2 (wood)	215	185	1 : 0.86	185	196	1 : 1.06
TF3 (cotton)	213	152	1 : 0.71	176	192	1 : 1.09
TF4 (polyurethane)	62	31	1 : 0.50	248	241	1 : 0.97
TF5 (*n*-heptane)	78	42	1 : 0.54	248	255	1 : 1.03

The results show that for the same fuel or fire source, the detector response in high-altitude areas will become complicated according to different combustion conditions. Especially for a flaming fire, as in the case of TF4 and TF5, the increase in speed was lessened at high altitudes compared to smouldering fires. Because many smoke detectors are using trend or threshold algorithms for fire alarms [20], the sensitivity of the alarm algorithm must be adjusted to fit the fire detection under low air pressure conditions.

**Figure 10.**
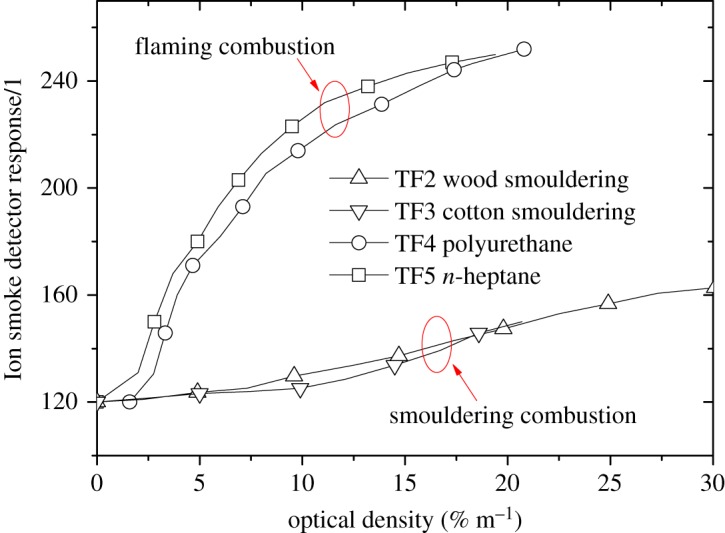
Correlation of Ion smoke detector signals with optical density for four test fires.

**Figure 11.**
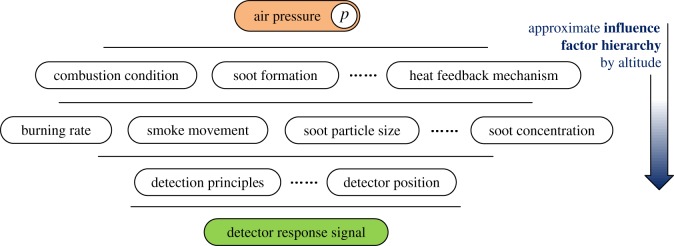
Hierarchical diagram of the effects of altitude on fire burning characteristics and detector responses.

Furthermore, based on the method of Litton [21], which determines a flaming or smouldering fire using an ionization chamber and light scattering in actual fire detection scenarios, we investigated the relationship between Ion smoke detector signals and optical densities using a smoke concentration meter, as shown in figure 10. Considering the smaller soot particle diameter distribution generated by smouldering fires mentioned above, the collision area for ionized air particles would also be smaller than that for smoke soot from flaming fires with the same concentration, leading to weaker Ion smoke detector response signals for smouldering combustion. Finally, a hierarchical diagram summarizing the relevant studies of the effects of high altitude on burning characteristics and detector responses is shown in figure 11 to give a better understanding based on physical causality. The diagram in figure 11 illustrates the mechanisms that determine how altitude affects the fire burning and smoke detector responses.

## 4. Conclusion

Comparative experimental studies on the influence of altitude on typical combustible burning and the related response signals of point-type Pho and Ion smoke detectors were conducted at two locations with four kinds of fuel sources, including wood, cotton, polyurethane and *n*-heptane, based on EN54 standards. The major conclusions are summarized as follows:

1. The influence of altitude on mass loss and burning rate for smouldering fires (TF2 and TF3) was shown to not be significant due to limited oxygen consumption, which is much smaller than that in flaming combustion processes.
2. The response signal tendency for Pho smoke detectors showed similar trends, with the burning rate in the TF4 and TF5 tests attributed to large heat release and strong turbulent buoyancy in flaming combustion, resulting in rapid smoke movement in the compartments. With the decreased burning rate for flaming fires, the signal was also weakened obviously at high altitude.
3. On the contrary, the altitude effect on Ion smoke detectors was found to be less significant than that on Pho smoke detectors. A mechanism analysis was proposed to investigate this phenomenon, which was based on the detection principle of the ionization chamber.
4. The average increases in speeds and peak values of detector response signals for each test were compared to provide a quantitative reference point for detector parameter or algorithm design and fire protection engineering at high altitudes.
5. The relationship between ionization chamber signals and smoke optical densities by the combined utilization of an Ion smoke detector and smoke concentration meter was proposed, which showed different response rules in smouldering and flaming fires.

Moreover, an approximate hierarchical diagram was introduced to provide a better understanding of the altitude and pressure effects on burning behaviour and the related response signals of detectors.

## Supplementary Material

Comparison of TF2; Comparison of TF3; Comparison of the TF4; Comparison of the TF5

## Data accessibility

We uploaded the experimental data as electronic supplementary material.
